# An In Silico and In Vitro Assessment of the Neurotoxicity of Mefloquine

**DOI:** 10.3390/biomedicines12030505

**Published:** 2024-02-23

**Authors:** Basma M. El Sharazly, Abrar Ahmed, Hany M. Elsheikha, Wayne G. Carter

**Affiliations:** 1 Parasitology Department, Faculty of Medicine, Tanta University, Tanta 31527, Egypt; basma.mohamed@med.tanta.edu.eg; 2 Clinical Toxicology Research Group, School of Medicine, Royal Derby Hospital Centre, University of Nottingham, Derby DE22 3DT, UK; 3 Faculty of Pharmacy, Punjab University College of Pharmacy, University of the Punjab, Lahore 54590, Pakistan; abrar.pharmacy@pu.edu.pk; 4 School of Veterinary Medicine and Science, University of Nottingham, Sutton Bonington LE12 5RD, UK; hany.elsheikha@nottingham.ac.uk

**Keywords:** antimalarial drug, cholinesterase inhibitor, mefloquine, neurotoxicity, redox stress

## Abstract

Mefloquine (MQ) is a quinoline-based anti-malarial drug used for chemoprophylaxis or as a treatment in combination with artesunate. Although MQ has clear anti-*Plasmodium falciparum* properties, it can induce neurotoxicity and undesired neuropsychiatric side effects in humans. Hence, this study aimed to characterize the neurotoxicity of MQ using human neuroblastoma SH-SY5Y cells. The effects of MQ on neuronal toxicity and cell viability were investigated over a concentration range of 1–100 µM using 3-(4,5-dimethylthiazol-2-yl)-2,5-diphenyltetrazolium bromide (MTT) and lactate dehydrogenase (LDH) assays. The influence of MQ on cellular bioenergetics was examined by measuring cellular ATP levels and from the induction of reactive oxygen species (ROS). An in silico approach was used to assess the potential neurotoxicity of MQ mediated via binding to the active sites of acetylcholinesterase (AChE) and butyrylcholinesterase (BuChE) and then experimentally validated via in vitro enzymatic assays. MQ was cytotoxic to neuronal cells in a concentration and exposure duration dependent manner and induced a significant reduction in viability at concentrations of ≥25 µM after a 24 h exposure. MQ adversely impacted cellular bioenergetics and significantly depleted ATP production at concentrations of ≥1 µM after 24 h. MQ-induced cellular ROS production, which was correlated with the induction of apoptosis, as revealed by flow cytometry. In silico studies suggested that MQ was a dual cholinesterase inhibitor and one with remarkably potent binding to BuChE. Modelling data were supported by in vitro studies which showed that MQ inhibited both human AChE and BuChE enzymes. In summary, MQ is an antimalarial drug that may induce neurotoxicity by impacting cellular bioenergetics and perturbing the activity of cholinesterases at exposure concentrations relevant to human dosage.

## 1. Introduction

Although there has been a recent decline in the incidence of malaria, there were still an estimated 229 million global malaria cases in 2019 in the 87 malaria-endemic countries [1]. Likewise, the number of deaths attributed to malaria declined from 736,000 in 2000 to 409,000 in 2019 [1], but treatment improvements are needed to reduce this number further.

Mefloquine (MQ) is a synthetic 4-quinolinemethanol, a derivative of quinine that is used as an antimalarial drug [2]. MQ is listed as a WHO essential medicine used for chemoprotection and in combination with artesunate as a treatment for *Plasmodium falciparum* malaria [3]. MQ is taken orally, once weekly for chemoprophylaxis, beginning 2–3 weeks before entering an area endemic for malaria, and then dosing continued for 4 weeks after leaving [4]. MQ is an effective treatment for mild to moderate cases of malaria caused by *P. falciparum* and for chloroquine-resistant forms of *P. falciparum* [5]. Indeed, MQ in combination with artesunate outperforms MQ alone for treating uncomplicated *P. falciparum* malaria in regions with low malaria transmission [6].

The antimalarial activity of MQ (either as a sole pharmacotherapy or in conjunction with co-administered drugs) reflects several mechanisms by which MQ exerts its toxicity against *P. falciparum*. These include the action of MQ as an inhibitor of protein synthesis by targeting the 80S ribosome [7]; interaction with heme and the formation of a toxic byproduct [8]; disruption of parasite endocytosis [9]; generation of parasitic redox stress [10]; interaction with membrane phospholipids [11] and via competitive inhibition of fatty acyl-CoA binding to acyl-CoA binding proteins limiting parasitic growth and proliferation [12]. 

The more common side effects that can arise from the use of MQ include headache, nausea, diarrhea, dizziness, and skin reactions; additionally, there are a number of neuropsychiatric reactions some of which can be serious, including anxiety, insomnia, seizures, depression, psychosis, and suicidal ideation [4]. Hence, contra-indication guidance for MQ usage includes an avoidance of prophylaxis if a patient has a history of psychiatric disorders (including depression) or convulsions [4]. The likelihood of the induction of neuropsychiatric side effects from MQ may increase when it is taken at relatively high and/or repeated doses [13,14]. In addition, MQ therapy can also induce gastrointestinal side effects and elevations in serum aminotransferases associated with acute liver injury [13]. 

MQ usage may induce cytotoxicity through several cellular phenomena including cytoplasmic signalling effects, as well as the accumulation of MQ within lysosomes, endoplasmic reticulum (ER), and mitochondria, and these can collectively lead to cell cycle deregulation and cell death [15]. However, there is still an incomplete understanding of the causal mechanisms behind its neurological and psychiatric effects. Several mechanisms have been proposed, and these include the impact of MQ on neurotransmitters through the weak or moderate inhibition of acetylcholinesterase (AChE) [16,17], which may also enhance the release of the neurotransmitter, gamma-aminobutyric acid (GABA), in part mediated by extracellular Ca^2+^ levels [18]. 

In addition to the reports of neuropsychiatric adverse events after taking MQ [19], other neurotoxic effects of MQ have been documented [20]. These include an in vitro report that MQ can block gap junctional coupling between interneurons [21], MQ effects on Ca^2+^ homeostasis and the induction of ER stress [22]; the inhibition of ATP-sensitive potassium (K_ATP_) channels [23]; the induction of cellular redox stress and synaptodendritic degeneration [24].

Since MQ administration can induce neuropsychiatric effects [4,13,14,19], it is important to establish the exposure threshold to MQ that is toxic to neuronal cells and further consider the mechanisms that may contribute to these neurotoxic side effects. Hence, in this study SH-SY5Y neuroblastoma cells were treated with MQ over a broad concentration and exposure duration range and the effects of MQ on cell viability and cellular bioenergetics were examined. In addition, the ability of MQ to act as a specific cholinesterase inhibitor was assessed using both in silico and in vitro approaches.

## 2. Materials and Methods

### 2.1. Chemicals and Reagents

All chemicals were obtained from Sigma-Aldrich (Poole, UK) unless otherwise specified. Mefloquine hydrochloride (M2319, Sigma-Aldrich, Poole, UK) (R*,S*)-(+/−)-α-2-Piperidyl-2,8-bis(trifluoromethyl)quinoline-4-methanol monohydrochloride (MW 414.77) was prepared as a 50 mM stock in dimethyl sulphoxide (DMSO) and diluted in SH-SY5Y cell culture media prior to application to cells.

### 2.2. Cytotoxicity Assays

The SH-SY5Y neuroblastoma cell line, originally purchased from the European Collection of Authenticated Cell Culture (ECACC) (ECACC-94030304) was used to test the cytotoxic effects of MQ using a 3-(4,5-dimethylthiazol-2-yl)-2,5-diphenyltetrazolium bromide (MTT) assay [25]. SH-SY5Y cells (passage #14) were seeded in 96-well plastic tissue culture plates at a density of 3 × 10^4^ cells/well in media composed of 43.5% Eagle’s Minimum Essential Medium (EMEM) supplemented with 43.5% Ham’s F12 nutrient mix, 1% MEM Non-Essential Amino Acid Solution (NEAA), 10% heat-inactivated Foetal Bovine Serum (FBS), 1% penicillin-streptomycin solution containing 10 mg/mL streptomycin and 10,000 IU penicillium and 2 mM glutamine. Cells were cultivated at 37 °C with an atmosphere of 5% CO_2_ and 95% humidity until they were 80–85% confluent, and then MQ was added to the media at concentrations of 1, 10, 25, 50, and 100 µM. After 6, 24, and 48 h of incubation at 37 °C, the cell culture media was removed and replaced with new growth media containing 0.5% (*w*/*v*) MTT. After two hours of incubation at 37 °C, the media was removed from the plates and replaced with DMSO, before the absorbance was read at 570 nm using a Varioskan™ LUX multimode microplate reader (ThermoFisher Scientific, Waltham, MA, USA) to quantify the formation of formazan. At these cell seeding and growth conditions, the MTT signal displays linearity [26]. The viability of vehicle control treated cells was set at 100%.

A lactate dehydrogenase (LDH) assay was undertaken as an alternative method to validate changes in cell viability. Extracellular LDH activity, released due to a loss of cell membrane integrity was measured using a CyQUANT, LDH cytotoxicity assay kit (ThermoFisher Scientific, Waltham, MA, USA) according to the manufacturer’s guidelines. SH-SY5Y cells were seeded as described above for MTT assays and treated with MQ in cell culture media at concentrations of 0.1, 1, 10, 25, 50, and 100 µM for 6, 24, and 48 h. Fifty microlitres of spent media from each treatment was removed and allowed to react with the LDH reaction mix for 30 min at room temperature protected from light before adding 50 µL of stop solution to each sample well. The final product was measured spectrophotometrically at 490 nm and 680 nm using a Varioskan™ LUX multimode microplate reader (ThermoFisher Scientific, Waltham, MA, USA). The reading taken at 680 nm absorbance value was subtracted from the 490 nm absorbance value to calculate LDH activity as previously described [27]. The OD values for each of the treatments were normalised to the mean of the negative control, and the percentage of LDH production was determined. 

### 2.3. Cellular Bioenergetics Assay

An ATP assay was used to examine the potential effect of MQ on cellular bioenergetics. SH-SY5Y cells were grown in 6-well plates and MQ was applied at concentrations of 0.1, 1, 10, 15, 20, 25, 50, and 100 µM in cell culture media. Following the same durations of MQ treatment as described above in Section 2.2, plates were washed three times with ice-cold phosphate buffered saline (PBS) and then with 500 µL of Tris base/ethylenediaminetetraacetic acid (EDTA) buffer (100 mM Tris and 4 mM EDTA, pH adjusted to 7.7) via agitation on ice for 5 min, before scraping the cells into the buffer. Incubation at 100 °C for 10 min was carried out after adding 9 volumes of boiling Tris/EDTA buffer to the extracted cell suspension. Following centrifugation at 1000× *g*, samples were kept on ice until required. The ATP levels were measured using the ATP Bioluminescence Assay Kit CLS II (11 699 695 001, Roche, Basel, Switzerland) according to the manufacturer’s guidelines. In brief, ATP standards were prepared at a concentration range of 1 × 10^−4^ to 1 × 10^−10^ moles and prepared samples or standards were added to white, opaque 96-well plates (Corning Incorporated, San Diego, CA, USA). After adding luciferase reagent to each well, a Varioskan™ LUX multimode microplate reader (ThermoFisher Scientific, Waltham, MA, USA) was used to measure the luminescence at an integration time of one second. Based on the ATP standard curve, the ATP content in control and treated samples was interpolated. Each experiment was conducted in triplicate, with blank values subtracted from the average. For each treatment, corrected luminometric readings were normalised to the control mean so that ATP levels could be expressed as a percentage of that value [27].

### 2.4. Mitochondrial Membrane Potential (MMP) Assay

The mitochondrial membrane potential (MMP) was investigated using a MitoTracker Green FM probe (M-7514, ThermoFisher Scientific, Waltham, MA, USA) that can passively diffuse across the plasma membrane to label active mitochondria. SH-SY5Y cells (3 × 10^3^ cells/well) were treated with MQ at concentrations of 0.1, 0.4, 1.1, 3.3, and 10 µM in a 96-well plate for 6, 24, and 48 h. After the incubation period with MQ, 50 nM MitoTracker Green FM dye was added to each well and incubated for 30 min at 37 °C, and then the plates were resuspended in PBS [27]. Carbonyl cyanide 4-(trifluoromethoxy)phenylhydrazone (FCCP) (300 µM for 24 h) was used as a positive control for mitochondrial membrane uncoupling. The fluorescence signal was measured using a Varioskan™ LUX multimode microplate reader (ThermoFisher, Waltham, MA, USA) with excitation and emission spectra of 490 nm and 516 nm, respectively. The mean value of the MMP in the treated cells was calculated in comparison with that of the control cells. 

### 2.5. Measurement of Reactive Oxygen Species Generation

The levels of reactive oxygen species (ROS) were determined using a 2′,7′-dichlorofluorescein diacetate (DCFDA) assay. 2′,7′-dichlorodihydrofluorescein diacetate (DCHFDA) powder was dissolved in ethanol to prepare a 25 mg/mL stock solution and was stored at −20 °C until required. Before the assay, a concentration of 1 mM of DCHFDA was prepared in PBS. SH-SY5Y cells were seeded in black, clear-bottomed sterile 96-well plates (Corning Incorporated, Corning, NY, USA) as detailed in Section 2.2. Cells were then treated with MQ at concentrations of 0.1, 0.4, 1.1, 3.3, and 10 µM in the presence or absence of 10 mM N-acetyl cysteine (NAC) and 50 µM DCHFDA. As a positive control for the induction of redox stress, H_2_O_2_ (500 µM) was added 30 min prior to the completion of the experiment along with 50 µM DCHFDA. After a 3- and 6-h incubation with MQ, plates were washed three times with PBS and the fluorescence signal from DCFDA was measured using a Varioskan™ LUX multimode microplate reader (ThermoFisher Scientific, Waltham, MA, USA), with excitation and emission spectra of 495 nm and 529 nm, respectively. The fluorescence measurements were adjusted to the mean of the control values after the subtraction of blanks. A percentage was calculated relative to the positive control to determine the levels of ROS generated.

### 2.6. Flow Cytometric Assessment of Cell Death

SH-SY5Y cells were plated at 6 × 10^5^ cells/well in 6-well plates and cultured for 48 h. To examine the effect of MQ on apoptotic cell death, cells were treated with MQ for 24 h and then stained using a FITC Annexin V/Dead Cell Apoptosis Kit (V13242, ThermoFisher Scientific, Waltham, MA, USA) according to the manufacturer’s instructions and incubated for 15 min. Apoptosis was detected using a CytoFlex S Flow Cytometer (Beckman Coulter Inc., Brea, CA, USA). A count of ≈10,000 events was collected for each sample and the percentage distribution of dead cells was calculated using Kaluza analysis software (version 2.1, Beckman Coulter Inc.) (https://www.mybeckman.uk/flow-cytometry/software/kaluza/downloads (accessed on 1 September 2022). The data presented are representative of three independent experiments.

### 2.7. In Silico Analysis

#### 2.7.1. Structure Preparation

The crystallographic structures of recombinant human acetylcholinesterase (AChE) (PDB Code:4EY5) and human butyrylcholinesterase (BuChE) (PDB Code: 6I0C) were obtained from the Protein Data Bank (PDB) and prepared using the Protein Preparation Wizard available in the Maestro v12.3 (Schrödinger, LLC, 2023) (New York, NY, USA), (https://www.schrodinger.com/downloads/releases) (accessed on 6 August 2023) software package. All missing residues were included and all ligands except cofactors and crystallographic water molecules were removed. H-bonds were adjusted at variable pH, assigned bond order and the structures were protonated according to a pH of 7.0. The protonated structures were minimised using an Optimised Potentials for Liquid Simulations (OPLS) force field.

#### 2.7.2. Ligand Preparation

Mefloquine was drawn using ChemDraw (available from https://www.perkinelmer.com/uk/product/chemoffice-chemoffice) (accessed on 6 August 2023) and prepared using the LigPrep module available in Maestro (Schrödinger, LLC, 2023, New York, NY, USA). An energy minimisation was carried out using the OPLS-2005 force field.

#### 2.7.3. Molecular Docking

Molecular docking studies were performed using Glide with default parameters, available in Maestro (Glide, Schrödinger, NY, USA). First, a binding pocket was located using a receptor grid generation using key residues involved in ligand binding. Molecular docking (XP) calculations were performed using Glide at the binding site of the AChE and BuChE proteins with default parameters. No constraints were applied for all the docking studies. Multiple poses of MQ were attained after the docking process and the best docked pose was selected for the analysis.

#### 2.7.4. Prime Molecular Mechanics-Generalised Born and Surface Area Solvation (MM-GBSA) 

For the calculation of binding free energy (ΔGbind) of each ligand docking complex, prime MM-GBSA was applied using the following equation:

ΔGbind = ΔEMM + ΔGsolv + ΔGSA

where, ΔEMM is the difference in the minimised energy between the protein-inhibitor complex and the sum of energies of the unliganded protein and the ligands, and ΔGsolv is the difference in MM-GBSA. Solvation energy was applied to analyze the binding free energy decompositions of the ligand–protein complex and the sum of energies for the unliganded protein and the ligand. ΔGSA is the difference in surface area energies for the complex and the sum of the surface area energies for the protein and ligand when considered individually. 

The molecular dynamic simulation was performed based on the receptor-ligand complex obtained from molecular docking. The ligand poses were minimised using the local optimisation feature in Prime, OPLS-2005 force field and a Generalised-Born/Surface Area continuum solvent model was used to calculate the energies of each complex. The ligand strain energy was also considered during the simulation process.

#### 2.7.5. Molecular Dynamics Simulations

The estimation of stability and interaction of AChE or BuChE with MQ was achieved using the Maestro-Desmond v12.3 Schrödinger software package. A Molecular Dynamic (MD) simulation model was constructed by harnessing a Desmond System Builder. Water molecules were added to the system. The protein–ligand complex was kept in an orthorhombic box shape and placed in the centre of the box by minimising the volume in the system builder. The charge of each system was neutralised by the addition of Na^+^ or Cl^−^ ions, and then the system was minimised and pre-equilibrated by using force field Optimised Potentials for Liquid Simulations (OPLS3e), as this produces greater accuracy against performance benchmarks that assess small molecule conformational propensities, solvation, and protein–ligand binding. Each MD simulation was run for 150 ns at a normal pressure and temperature (NPT) ensemble of 300 K temperature and 1.013 bars pressure. The system was set to a relaxed state, with protein and ligand structural properties, Root Mean Square Deviation (RMSD) of the ligand–protein, and the Root Mean Square Fluctuation (RMSF) for interacting residues with the ligand performed as described previously [28]. 

### 2.8. Cholinesterase (ChE) Activity Assessments

Based on the original method of Ellman et al. (1961) [29], human acetylcholinesterase (AChE) and butyrylcholinesterase (BuChE) activities were quantified in a 96-well microtiter plate. Ten microlitres of MQ (over a concentration range of 1, 10, 25, 50, and 100 µM) was mixed with 3 µL of 1 U/mL human AChE enzyme (recombinant) (C1682, Sigma-Aldrich, Poole, UK) or 10 µL of 2 U/mL human BuChE enzyme (B4186, Sigma-Aldrich, Poole, UK), 150 µL of 0.38 mM 5,5-dithio-bis-(2-nitrobenzoic acid) (DTNB) and 43 µL of PBS, pH 7.4. After incubation at room temperature for 20 min, 4 µL of 35 mM acetylthiocholine iodide (ATCI) (substrate for AChE) or butyrylthiocholine iodide (BTCI) (substrate for BuChE) was added, respectively, and the absorbance read at 412 nm every 30 s for 5 min using a Varioskan LUX multimode microplate reader (ThermoFisher Scientific, Waltham, MA, USA). Reagent blanks were taken without AChE or BuChE. Positive control inhibition of human AChE and BuChE was undertaken with 10 µM malaoxon (Chem Service Inc., West Chester, PA, USA) [30], or 10 mM ethopropazine hydrochloride (E5406, Sigma-Aldrich, Poole, UK) [31], respectively. The percentage activity of AChE or BuChE after MQ incubations was calculated relative to the negative control (the enzyme only) in the absence of MQ or the inhibitor and was defined as 100% enzymatic activity. Three independent experiments were conducted, with each assay data point performed in duplicate, from which a mean was calculated.

### 2.9. Statistical Analysis

The statistical software PRISM 9 was used for all data analysis (GraphPad Software Inc., San Diego, CA, USA). The data from several groups were compared using either one-way or two-way ANOVA tests with either Dunnett’s or Tukey’s multiple comparisons post-tests. The results for each control and treatment group were presented as means ± SEM. The threshold for statistical significance was set at *p* < 0.05. 

## 3. Results

### 3.1. Assessment of MQ Cytotoxicity

**Figure 1:**
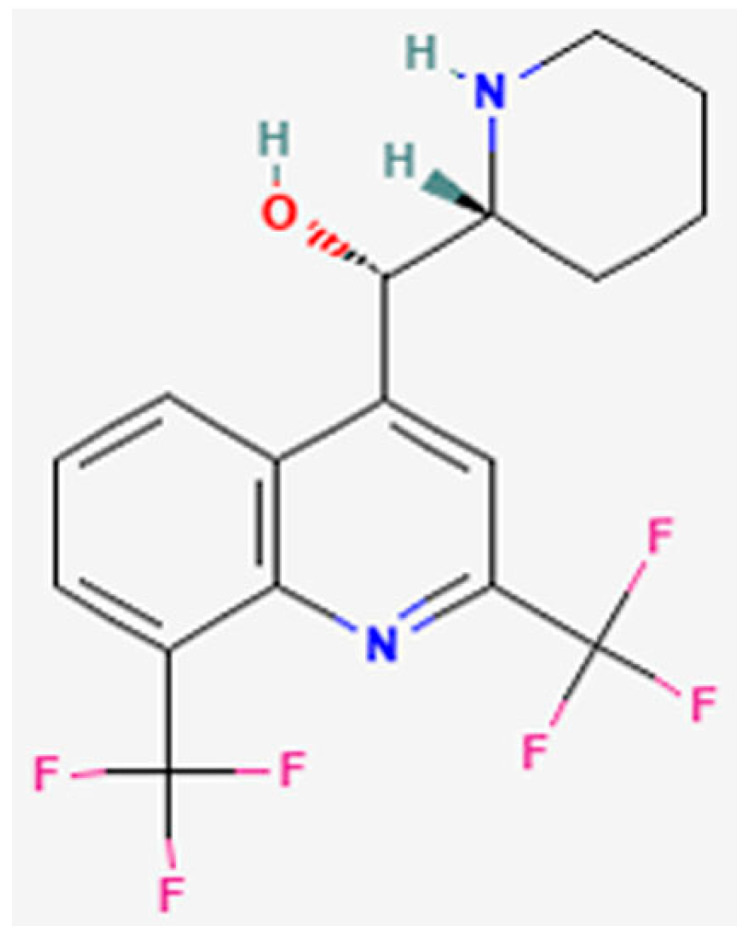
Chemical-Structure-of-Mefloquine.

**Figure 2:**
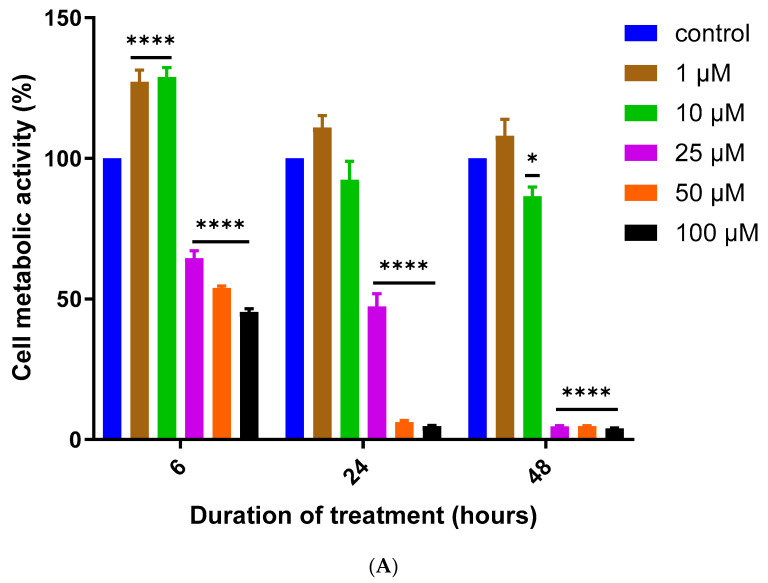

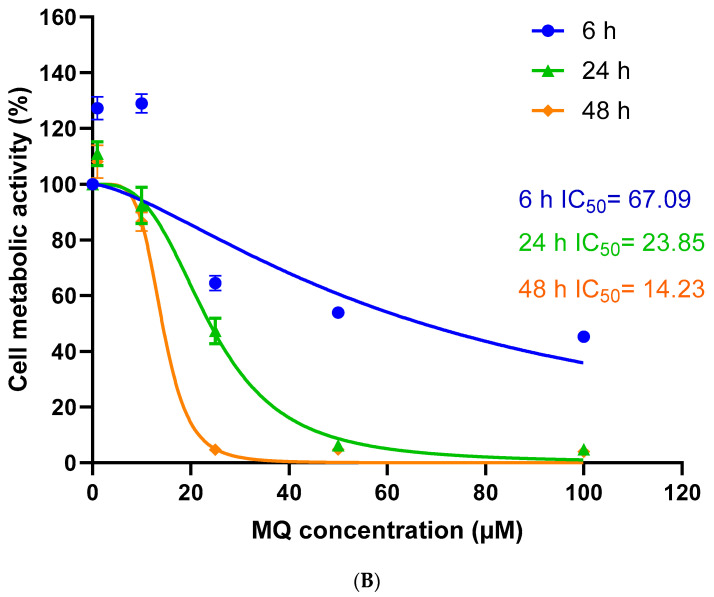
Mefloquine effects on cell metabolic activity as assessed using an MTT assay. (**A**) SH-SY5Y cells were treated with mefloquine (MQ) over a concentration range of 1–100 µM for 6, 24 and 48 h and cell metabolic activity quantified using an MTT assay. Readings were obtained from three individual experiments with triplicate assays performed for each data point. Results were analysed using a two-way ANOVA with Tukey’s multiple comparisons and are expressed as the mean ± standard error of the mean (SEM). For marked significance: * *p* < 0.05, **** *p* < 0.0001. (**B**) MTT readings were plotted using non-linear regression to provide an estimate of the concentration of MQ that produced 50% inhibition (IC_50_) of cell metabolic activity/viability.

The metabolic activity and cell viability of SH-SY5Y cells in response to exposure to MQ (Figure 1) [2] were monitored by an MTT assay at 6, 24, and 48 h as shown in Figure 2A,B. MQ primarily reduced cell metabolic activity in a concentration and exposure duration manner. However, 1 µM of MQ increased cell metabolic activity at all time points and this reached significance after 6 h (27%, *p* < 0.0001). Incubation with 10 µM MQ also significantly increased cell metabolic activity by approximately 29% (*p* < 0.0001) after 6 h but significantly reduced it by 13.6% at 48 h (*p* = 0.024). 

MQ was toxic to the neuroblastoma cells that reflected exposure and duration dependence from a threshold of ≥25 µM at all time points examined (Figure 2A), as detailed in Table 1.

**Figure 3:**
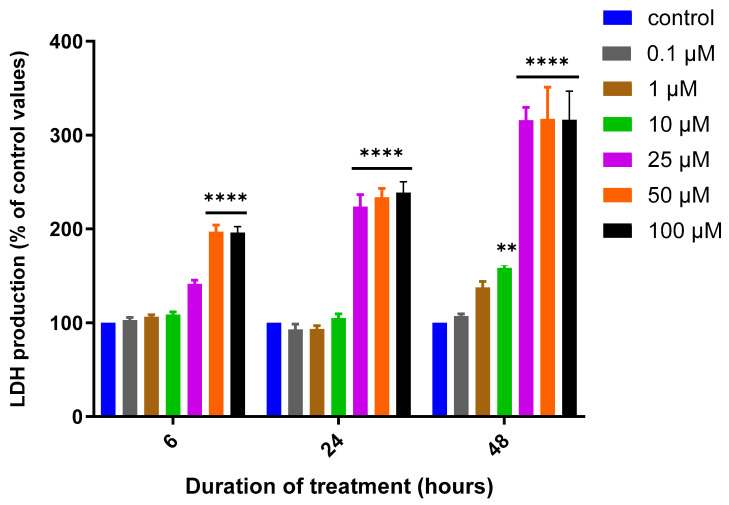
Mefloquine effects on cell viability assessed using an LDH assay. SH-SY5Y cells were treated with MQ over a concentration range of 0.1–100 µM for 6, 24 and 48 h and cell viability quantified using an LDH assay. Readings were obtained from three individual experiments with triplicates for each data point. Results were analysed using a two-way ANOVA with Tukey’s multiple comparisons test and expressed as the mean ± standard error of the mean (SEM). For marked significance; ** *p* < 0.01, **** *p* < 0.0001.

A reduction in cell metabolic activity, as measured by the MTT assay, is often used as a surrogate for changes in cell viability [32]. However, to provide additional confirmation of the impact of MQ on SH-SY5Y cell viability, an LDH assay was employed. MQ induced cytotoxicity in a concentration and exposure duration-dependent manner, as shown in Figure 3 and Table 2. The cytotoxicity of MQ at the higher concentrations of 25, 50, and 100 µM showed a dramatic increase in extracellular LDH levels, but with no significant LDH production observed at the lower concentrations of 0.1 to 10 µM, except for the latter after 48 h.

**Figure 4:**
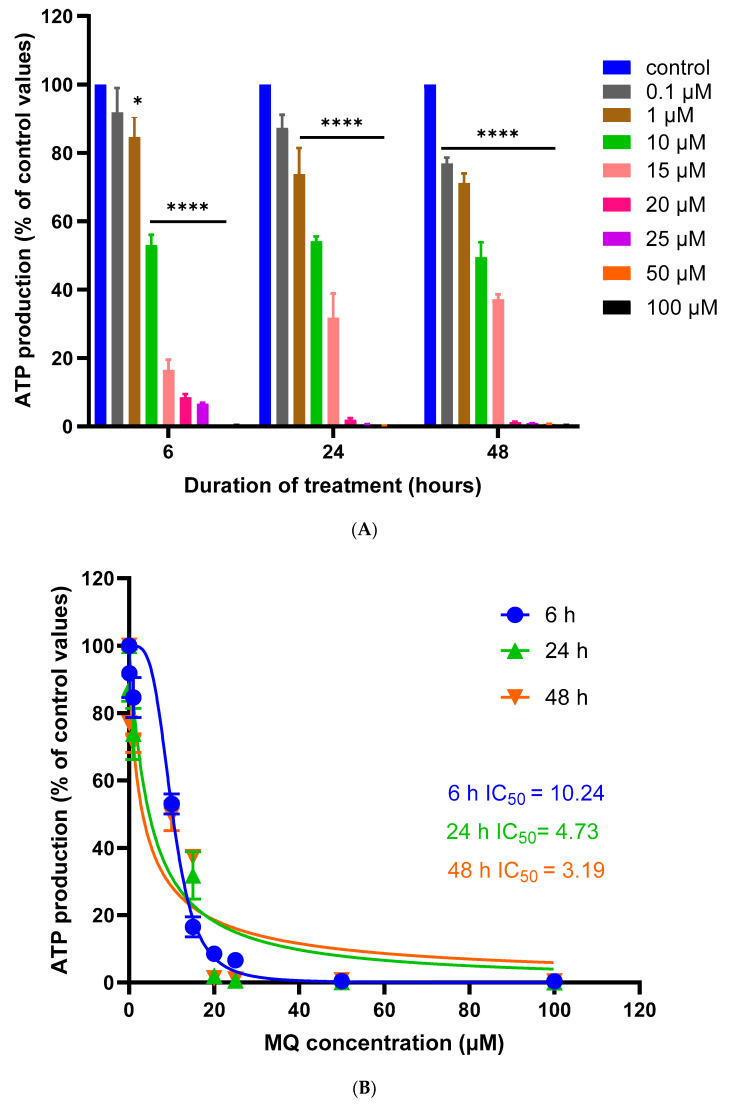
Mefloquine effects on cellular bioenergetics assessed using an ATP assay. (**A**) SH-SY5Y cells were treated with MQ over a concentration range of 0.1–100 µM for 6, 24 and 48 h and cellular ATP levels quantified. Readings were obtained from three individual experiments with triplicate measurements for each data point. Results were analysed using a two-way ANOVA with Tukey’s multiple comparisons test and expressed as the mean ± standard error of the mean (SEM). For marked significance; * *p* < 0.05, **** *p* < 0.0001. (**B**) ATP readings were plotted using non-linear regression to provide an estimate of the concentration of MQ that produced 50% inhibition (IC_50_) of the ATP assay.

MQ neurotoxicity and its impact on cellular bioenergetics and cell viability were also assessed by measurements of cellular ATP levels. MQ induced a concentration-dependent decrease in cellular ATP levels, and this also correlated with exposure duration (Figure 4A,B, and Table 3). Cellular ATP levels dropped significantly from 1 to 10 µM MQ after 6- and 24-h exposures, respectively, and from 0.1 µM MQ after 48 h. Hence, MQ impacted cellular bioenergetics with higher sensitivity to damage than that detected by MTT or LDH assays, and therefore, a lower IC_50_ concentration (Table 4). 

### 3.2. Effects of MQ on the MMP and Production of ROS

**Figure 5:**
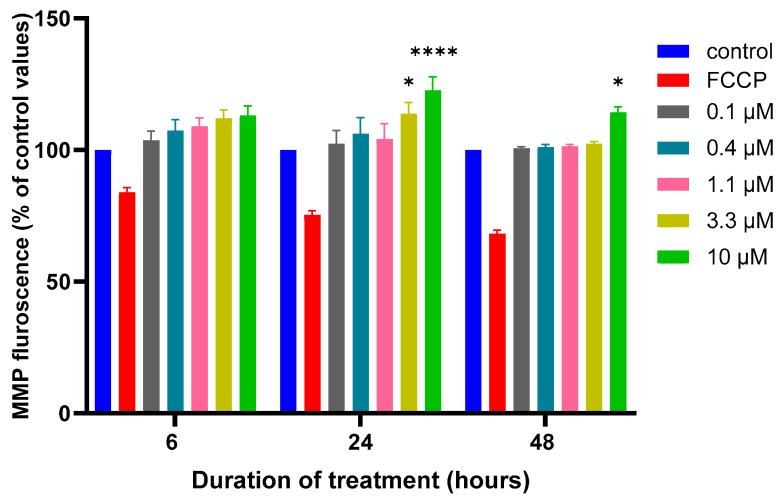
Effect of MQ exposure on the MMP. SH-SY5Y cells were treated with MQ at concentrations of 0.1, 0.4, 1.1, 3.3, and 10 µM for 6, 24, and 48 h and the MMP activity quantified. Readings were obtained from three individual experiments with triplicate measurements for each data point. Results were analysed using a two-way ANOVA with Tukey’s multiple comparisons and expressed as the mean ± standard error of the mean (SEM). FCCP was used as a membrane uncoupler. For marked significance; * *p* < 0.05, **** *p* < 0.0001.

An abnormality in the functionality of the MMP can impact the electron transport chain and limit the production of ATP. Hence, the effects of MQ on the MMP were examined using a Mitotracker green dye. Following a 6, 24, or 48 h exposure to MQ at concentrations of 0.1, 0.4, 1.1, 3.3, and 10 µM, the mean fluorescence values of the MMP were quantified (Figure 5). A concentration response was evident which reached significance at 3.3 and 10 µM after 24 h, and 10 µM after 48 h (Figure 5).

**Figure 6:**
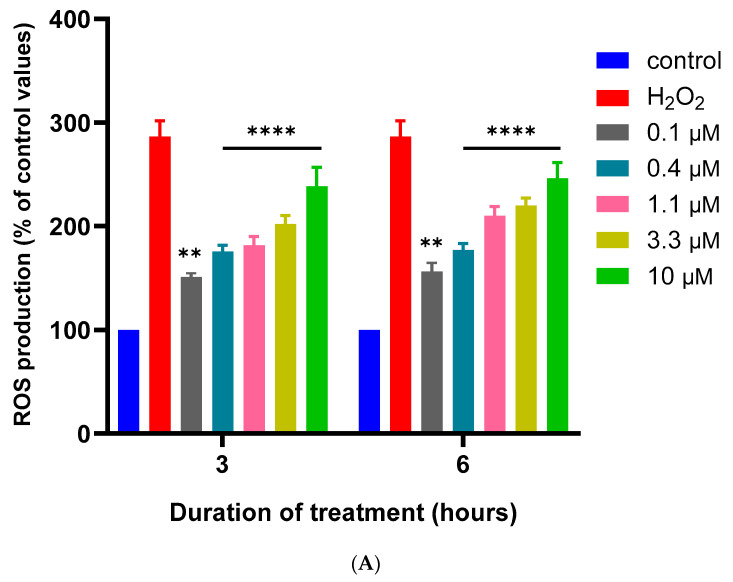

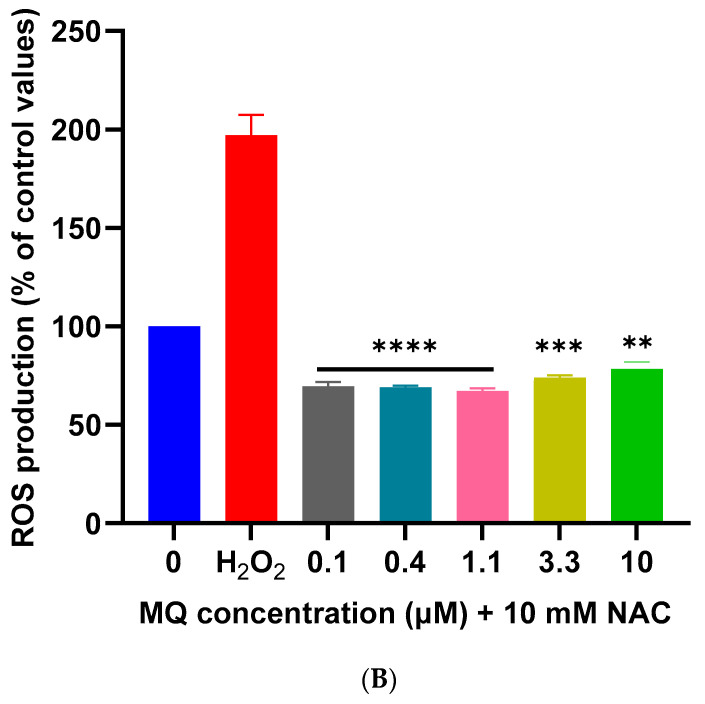
MQ induction of ROS quantified using a DCFDA assay. SH-SY5Y cells were treated with MQ at concentrations of 0.1, 0.4, 1.1, 3.3, and 10 µM and the levels of ROS quantified after 3 and 6 h using a DCFDA assay in the absence of NAC (**A**) or for 3 h in the presence of 10 mM NAC (**B**). Readings were obtained from three individual experiments with triplicate measurements for each data point. Results were analysed using a two-way ANOVA with Tukey’s multiple comparisons and expressed as the mean ± standard error of the mean (SEM). H_2_O_2_ was used as a positive control for the induction of ROS. For significance; ** *p* < 0.01, *** *p* < 0.001, **** *p* < 0.0001.

The effects of MQ on the production of intracellular ROS were quantified after 3 and 6 h using a DCFDA assay. MQ induced an increase in ROS production at both time points that was positively correlated with MQ concentration from the lowest tested concentration of 0.1 µM (Figure 6A). To confirm the induction of cellular ROS and oxidative stress, the co-treatment of cells with MQ (0.1–10 µM) and the antioxidant N-acetylcysteine (NAC) (10 mM), significantly inhibited MQ-induced ROS production after 3 h (Figure 6B). 

### 3.3. MQ-Induced Apoptosis

**Figure 7:**
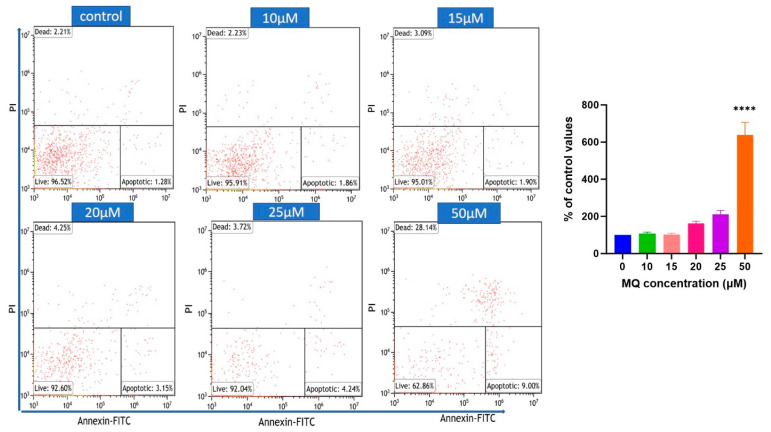

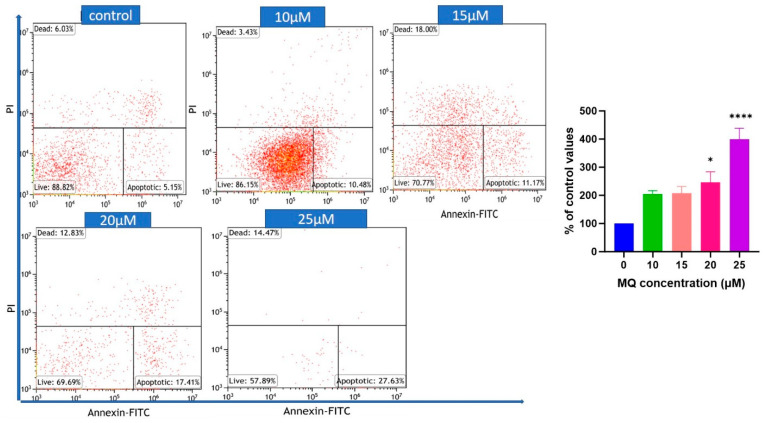
Apoptosis detection by flow cytometry using Annexin V-FITC/PI staining. SHSY5Y cells were treated with MQ (0–50 µM) for 6 h (**upper panel**) and 24 h (**lower panel**) and then stained with Annexin V-FITC/PI and the relative number of apoptotic cells quantified. Data represent the mean ± SEM of three independent experiments. For significance, * *p* < 0.05, **** *p* < 0.0001.

In accordance with the cell viability assay results, MQ induced a concentration- and duration-dependent increase in the number of apoptotic cells, and this reached significance at 50 µM MQ after a 6 h exposure (Figure 7), and 20 µM after a 24 h exposure (Figure 7).

### 3.4. In Silico Assessment of Cholinesterase Inhibitor Potential

#### 3.4.1. Molecular Docking

Molecular docking analysis was performed to provide insight into the potential of MQ to act as an inhibitor of AChE or BuChE. A prediction of the binding interactions between MQ and the target proteins AChE and BuChE was undertaken from which a docking score was generated. This represents the strength of the predicted binding between the compound and the target protein. Docking scores of MQ to AChE (−8.111 kCal/mol) were not as strong as those predicted for BuChE (−9.755 kCal/mol), and likewise, the MQ-BuChE also had a considerably lower minimal glide energy (−36.623 kCal/mol) compared to MQ-AChE (−7.018 kCal/mol), as shown in Table 5. 

**Figure 8:**
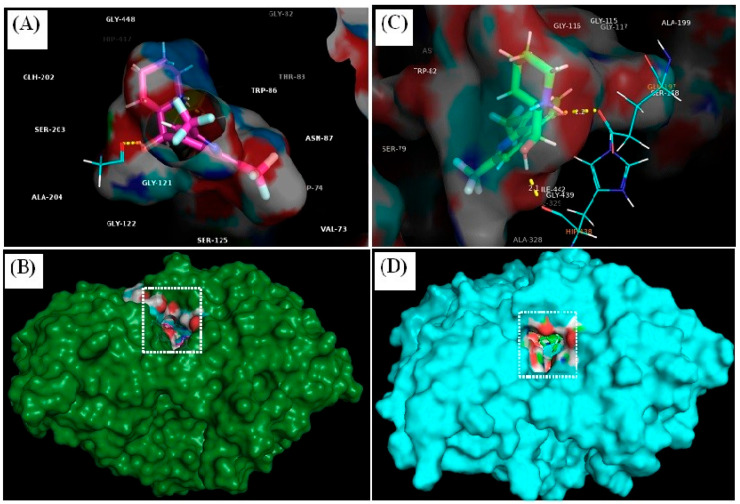
Mefloquine interactions with active site residues of acetylcholinesterase and butyrylcholinesterase. MQ binding with acetylcholinesterase (**A**) and with butyrylcholinesterase (**C**), with the drug molecule embedded inside the binding pocket of AChE (green-coloured) (**B**) and BuChE (cyan-coloured) (**D**).

MQ had an improved structural stabilisation with BuChE than AChE by forming conventional hydrogen bonding, aromatic hydrogen bonding, salt bridge, π-cation interaction and π-π interactions with BuChE. MQ forms conventional hydrogen bonding as well as a salt-bridge with Glu-197 of BuChE at a distance of 2.2 Å and 3.27 Å, respectively, and MQ hydrogen bonds with Tyr-337 at 2.1 Å and Gly-120 at 2.4 Å with AChE. Trp-82 forms a π-cation interaction and π-π interactions with Phe-329, as shown in Figure 8.

Mefloquine forms a polar interaction with Gly-120 and Tyr-337 of AChE, and Glu-197 and His-438 of BuChE, and a number of non-polar interactions with amino acids within the binding pockets of each enzyme, as shown in Table 6. 

#### 3.4.2. Prime/MM–GBSA Simulation

Relative binding energy analyses of the ligand (MQ) binding to AChE or BuChE were considered using prime energy calculations and post-docking energy minimisation studies using Prime Molecular Mechanics-Generalised Born Surface Area (MM-GBSA) analyses. The ΔG-binding value for MQ was calculated as −49.43 kcal/mol with BuChE, which was indicative of the formation of a stable complex. By comparison, a ΔG-binding value of 1.37 kcal/mol was calculated for MQ binding to AChE (Table 7).

#### 3.4.3. MD-Simulations

**Figure 9:**
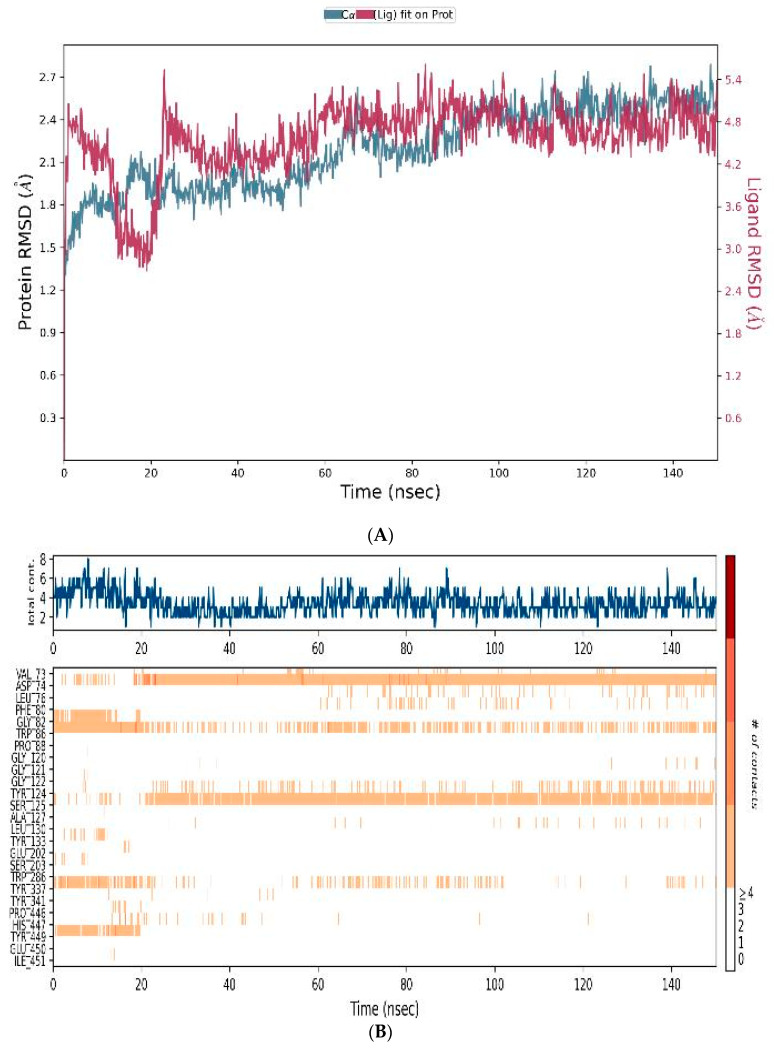

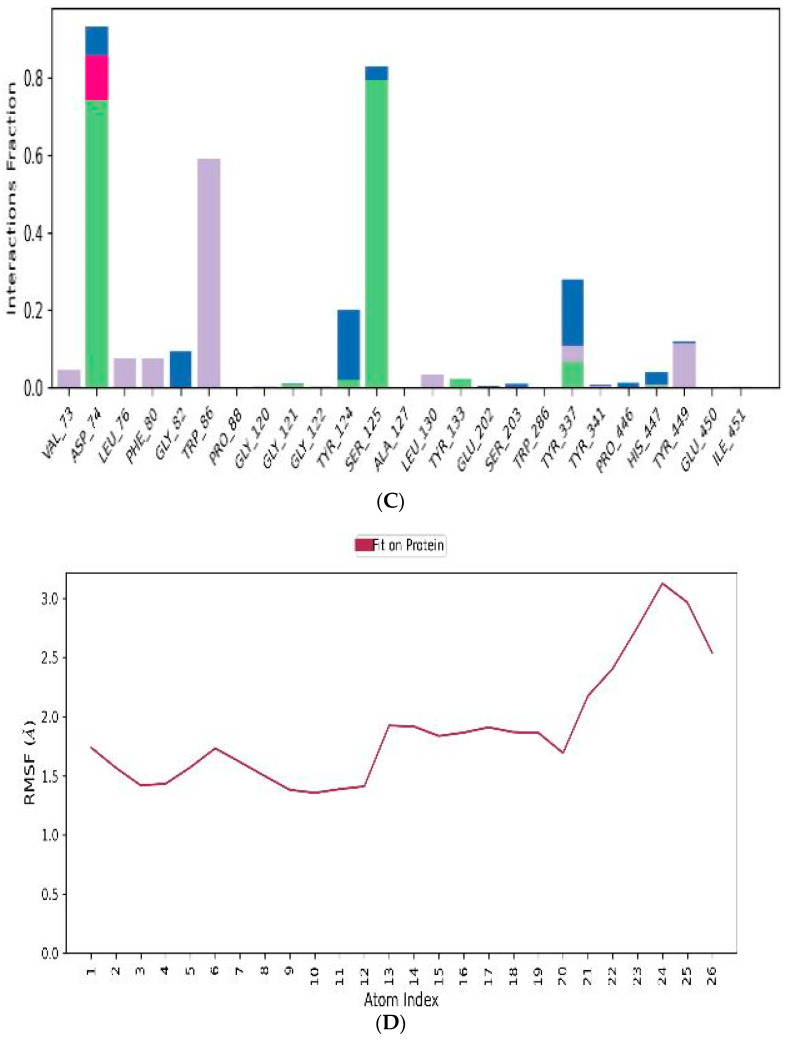

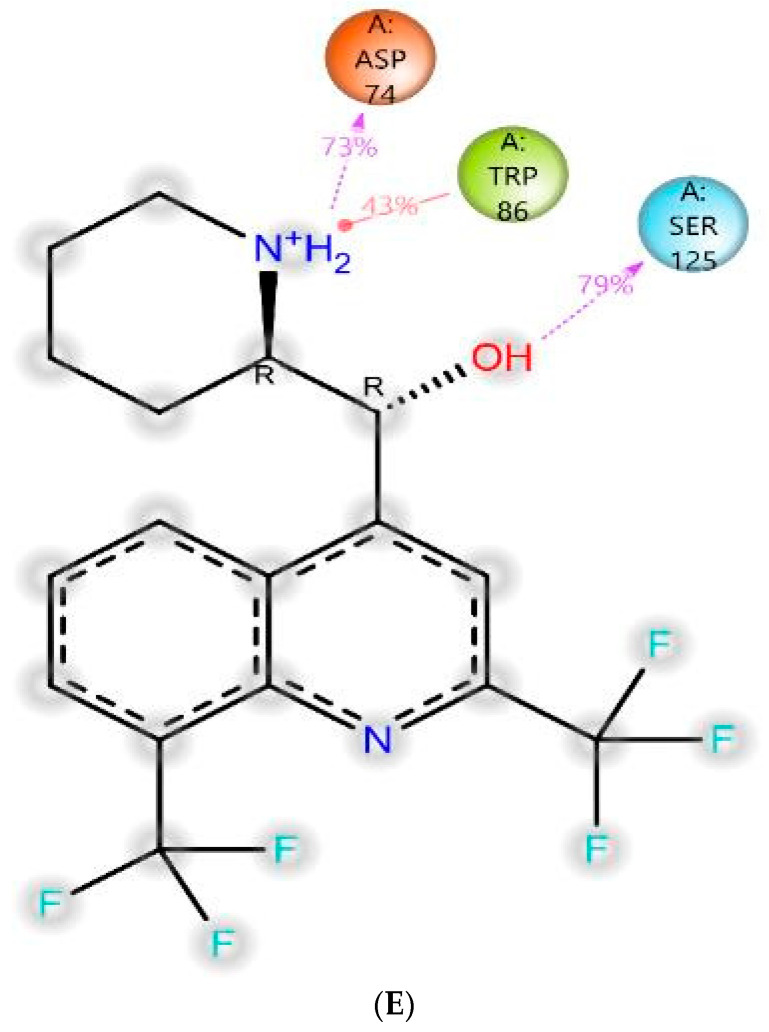
Molecular dynamics trajectory analysis of the mefloquine ligand complexed with AChE. (**A**) RMSD interaction of the protein (blue-colour) and ligand (red-colour). (**B**) Protein–ligand contacts. (**C**) Protein–ligand contacts histogram in which H-bond (green), hydrophobic (purple), ionic (pink), and water bridges (blue) are highlighted. (**D**) Ligand RMSF. (**E**) Ligand–protein interactions.

The formation of an MQ complex with AChE over a 150 ns MD simulation was undertaken to consider the binding dynamics of the ligand to the enzymatic active site. This assessed the stability and fluctuations of the ligand–protein complex within a simulated biological environment. Figure 9 depicts the MD trajectory data analysis of MQ. The RMSD plot (Figure 9A) indicated a stable ligand–protein complex throughout the entire simulation period. The RMSD plot showed a slight fluctuation between 0 and 20 ns but then remained stable throughout the remaining simulation time. The timeline of the protein–ligand interactions was plotted (Figure 9B) in which the top panel indicates the total number of specific protein–ligand contacts, and the bottom panel portrays the residue level interaction of the ligand. Overall, the ligand interacted well within the AChE binding pocket such that there was a minimum of two contacts present throughout the simulation period. The binding interactions between the ligand and active site amino acid residues inside the binding pocket of AChE were computed (Figure 9C). The RMSF plot of the ligand (Figure 9D) suggests that the ligand (MQ) is located within the binding pocket and interacts with residues Asp-74 and Ser-125 mainly through hydrogen bonding, and forms hydrophobic interactions with Val-73, Leu-76, Phe-80, Trp-86, Leu-130, and Tyr-449. In addition, water bridges with residues Gly-82, Tyr-124 and Tyr-337 also play a supportive role at the active site. The amide group of the MQ ligand interacts with the AChE active site via Asp-74 and Trp-86, whereas the hydroxyl group of MQ interacts with Ser-125 (Figure 9E).

**Figure 10:**
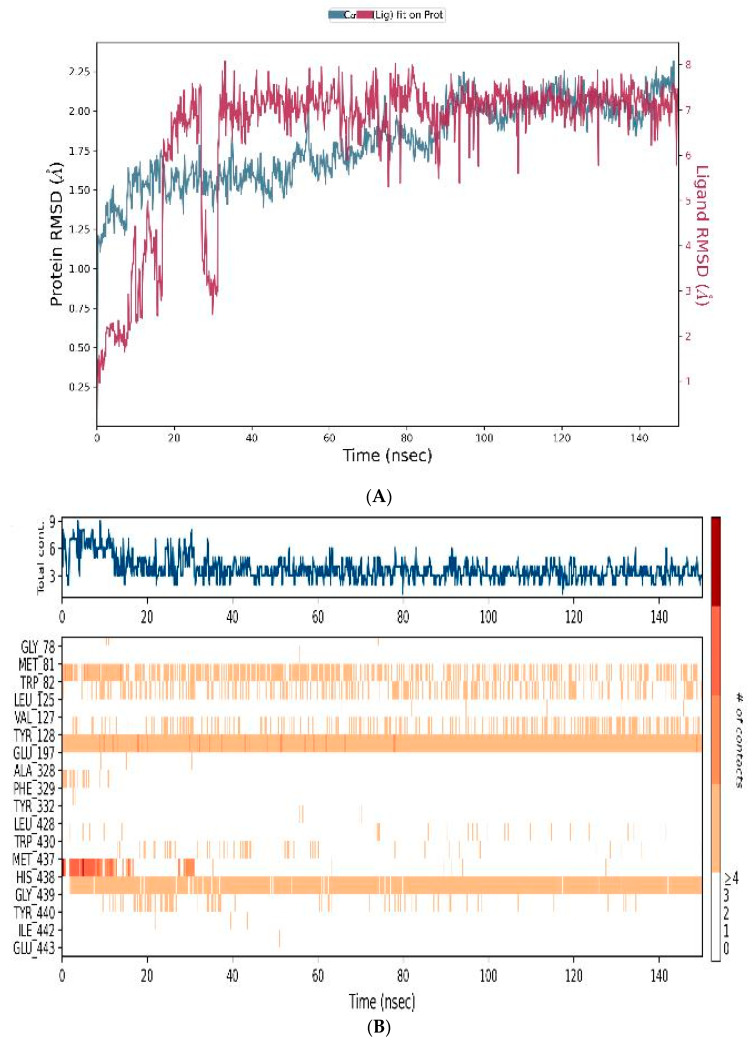

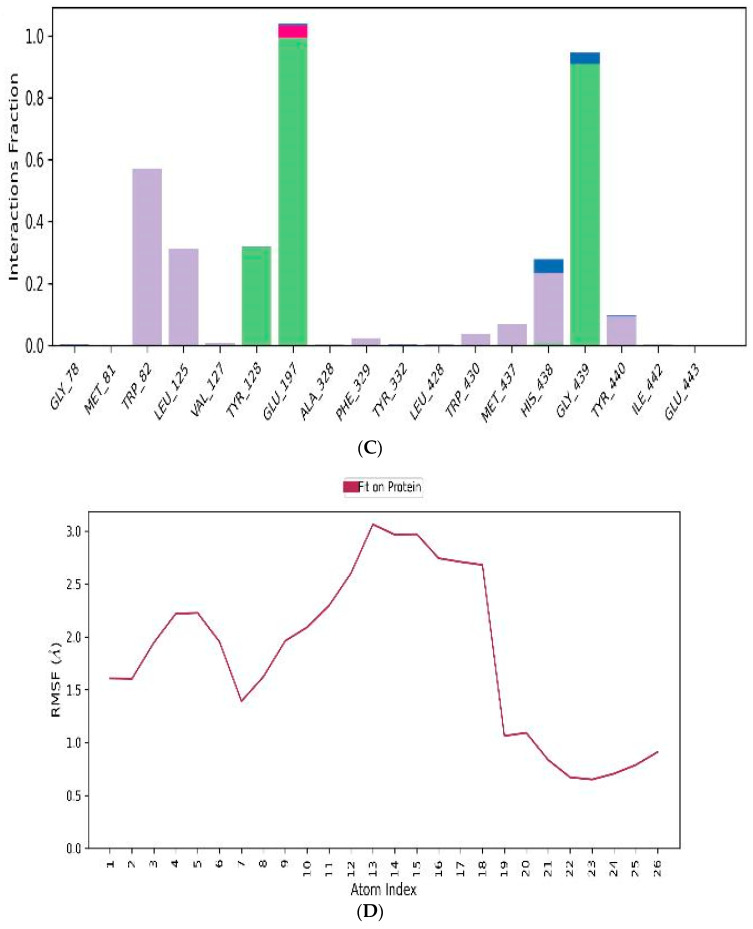

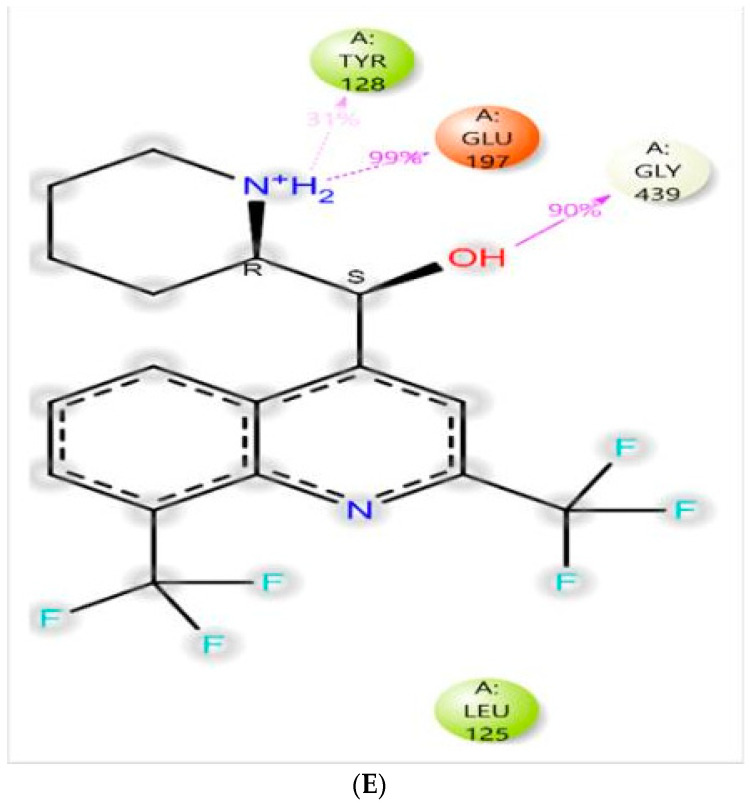
Molecular dynamics trajectory analysis of the mefloquine ligand complexed with BuChE. (**A**) RMSD interaction of the protein (blue-colour) and ligand (red-colour). (**B**) Protein–ligand contacts. (**C**) Protein–ligand contacts histogram in which H-bond (green), hydrophobic (purple), ionic (pink), and water bridges (blue) are highlighted. (**D**) Ligand RMSF. (**E**) Ligand–protein interaction.

The MD trajectory data analysis of MQ binding to BuChE is included as Figure 10. The RMSD plot indicated a generally stable ligand–protein complex during the simulation period. There was fluctuation between 0 and 80 ns and then a stable complex for the remaining simulation time (Figure 10A). The timeline of the protein–ligand interactions was plotted (Figure 10B) in which the top panel indicates the total number of specific protein–ligand contacts, and the bottom panel portrays residue level interaction of the ligand. The binding interactions between the ligand and active site amino acid residues inside the binding pocket of BuChE were computed (Figure 10C). The RMSF plot of the ligand (Figure 10D) suggested that the ligand is located within the binding pocket and bound to amino acid residues Trp-82, Leu-125, Tyr-332, Phe-329, Leu-428, Trp-430, Met-437, and Tyr-440 through hydrophobic interactions. Binding to Val-127, Tyr-128 and Gly-439 was conducted by hydrogen bonds, ionic bonds, and water bridges. The amide of the MQ ligand interacted with the BuChE active site via Tyr-128 and Glu-197, whereas the hydroxyl group of MQ interacted with Gly-439 (Figure 10E).

### 3.5. In Vitro Assessment of Cholinesterase Inhibitor Activity

**Figure 11:**
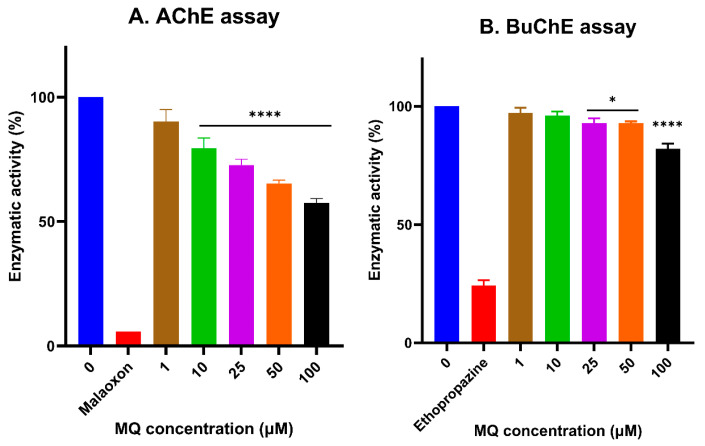
Cholinesterase inhibition by mefloquine. MQ over a concentration range of 1–100 µM was incubated with acetylcholinesterase (AChE) or butrylcholinesterase (BuChE) and the levels of AChE inhibition (**A**) or BuChE inhibition (**B**) quantified. Histograms represent means ± SEM from at least three independent experiments. For the positive control inhibitors, 10 µM malaoxon and 10 mM ethopropazine hydrochloride were used for AChE and BuChE, respectively. For marked significance * *p* < 0.05, **** *p* < 0.0001.

MQ inhibited AChE and BuChE activity in a concentration-dependent manner over the assayed concentration range of 1–100 µM (Figure 11A,B). Incubation with MQ triggered a significant reduction in AChE activity from 10 µM (Figure 11A) and from 25 µM for BuChE (Figure 11B). Interestingly, MQ induced a steady decline in AChE activity indicative of a concentration-response effect but the MQ inhibition of BuChE, although also detectable from 1 µM, was only limited to an approximately 10% reduction in the enzymatic activity at MQ exposures of between 1 and 25 µM (Figure 11A,B).

## 4. Discussion

The potential for drug toxicity and adverse reactions can limit the benefit of pharmacotherapy to treat human diseases. Despite often having intended specific target(s), drugs can cause side effects that can compromise their therapeutic efficacy, hence, the need for a comprehensive evaluation of drug toxicity. MQ is primarily utilised as an antimalarial chemoprophylactic drug or in combination with artesunate for anti-malarial therapy, but there are concerns with its potential for neurotoxicity and the induction of undesired neuropsychiatric manifestations [4,13,14,19,33]. Hence, herein, the neurotoxicity of MQ to neuroblastoma cells was examined over a broad concentration range of 0.1–100 µM and from 3 to 48 h. MQ was neurotoxic and significantly reduced cell viability and induced apoptosis from a threshold concentration of approximately 25 µM at 24 h and 10 µM at 48 h. Mitochondrial functionality was more sensitive to MQ exposures, with reduced ATP levels evident after a 24 h incubation with 0.1 µM MQ. The impact on cellular bioenergetics was detected with an altered MMP from 3.3 µM after 24 h and induction of ROS from 0.1 µM MQ exposures of 3- and 6-h. The neuropsychiatric effects of MQ could be mediated by impacting neurotransmitter levels and a combination of in silico and in vitro enzymatic assays suggested that MQ acts as a dual cholinesterase inhibitor, and furthermore, from modelling studies, displays potent binding affinity to BuChE. 

Neuroblastoma (SH-SY5Y) cells were used to evaluate MQ neurotoxicity since this human cell line is often employed for neurotoxicity assessment due to its homogeneous nature, and neuronal phenotype [34,35]. The effect of MQ on cell metabolic activity and cell viability was assessed using an MTT assay, which demonstrated both concentration and exposure duration dependence (Figure 2). An independent study considered the toxicity of MQ in SH-SY5Y cells by using an MTT assay and reported that MQ at 10 µM for 24 h reduced cell viability by approximately 20% [36], comparable to our studies. However, we extend the characterisation of MQ neurotoxicity by the generation of concentration-response curves and IC_50_ values for 6-, 24-, and 48-h exposures. These exposure times to MQ are in line with the pharmacokinetics of MQ in adults that have a peak concentration at 7 to 24 h in the blood after oral dosing [37,38]. 

To provide an alternative means to quantify cell viability, LDH assays were performed; these assays are reliant on the quantitation of extracellular LDH due to a loss of membrane integrity [39]. Similar to the MTT assays, the release of extracellular LDH followed both the concentration of MQ and the duration of its exposure (Figure 3), with a 24-h IC_50_ value of approximately 16 µM, comparable to that calculated from the MTT assays. 

The implications for neuronal damage and the loss of viability in response to exposure to MQ can be appraised by a consideration of therapeutic concentrations. The dosing of healthy subjects with MQ led to an approximate plasma concentration of MQ of 700 ng/mL (1.9 µM) [40], but prior to the clearance of parasitic infection, patients undertaking therapeutic regimens experienced higher blood concentrations of MQ of approximately 2800 ng/mL (7.4 µM) [40]. Similarly, patients treated for uncomplicated *P. falciparum* malaria with either of three mefloquine-artesunate (MQ-AS) formulations, displayed mean maximal MQ concentrations in whole blood of 2500–2820 ng/mL (6.6–7.5 µM), and with a terminal half-life of 14–15 days [41]. Likewise, in patients treated for acute *P. falciparum* malaria with MQ taken as a monotherapy, whole blood MQ concentrations peaked at close to 3000 ng/mL (7.9 µM), depending on the dosing regimen [38]. Hence, our experimental concentration range of 0.1–100 µM MQ covers the physiologically relevant concentrations for MQ taken for prophylaxis, blood concentrations of MQ when taken as an anti-malarial monotherapy or MQ-AS combination therapy, as well as supra-physiological and overdose concentrations. For the comparison of the latter, a whole blood concentration of 13.5 µM was associated with 1% mortality for the related 4-aminoquinoline drug, chloroquine [42]. Thus, a decline in neuronal cell number from approximately 10 µM (with a significant reduction at ≥25 µM) suggests that neuronal loss is unlikely for most patients who receive acute therapeutic dosing. However, since MQ crosses the blood–brain barrier, there is the potential for further toxicity due to the possibility of drug accumulation within brain tissue. 

ATP levels are also used as a surrogate for cell viability since lethal cell damage and a loss of membrane integrity results in an inability to synthesize ATP and a corresponding reduction in cellular ATP levels [43,44]. A decline in ATP levels can match the IC_50_ values generated by MTT and/or LDH assays in undifferentiated and differentiated SH-SY5Y cells in response to different neurotoxicants [27,32]. However, in our study, the sensitivity to MQ indicated notable potency for limiting mitochondrial function and blunting the generation of intracellular ATP (Figure 4), with a 24-h IC_50_ value of approximately 6 µM, lower than that estimated by MTT or LDH assays. In keeping with an impact on ATP production, disruption to the MMP was MQ concentration dependent 6 h post-exposure, with significant changes detected after exposures of 3.3 and 10 µM for 24 and 48 h, respectively (Figure 5). In support of the impact of MQ on mitochondria, recent studies have shown that 10 µM MQ reduces the cell number in KYSE150 cells (an oesophageal squamous cell carcinoma cell line), reduces the NAD^+^/NADH ratio, and also influences the mitochondrial proteome, including key proteins involved in the oxidative phosphorylation reactions that lead to ATP production [45]. 

There was also a concentration-dependent elevation of intracellular ROS at 3- and 6-h post-exposure (presumably liberated from damaged mitochondria) with a significant induction of ROS even at the lowest MQ concentration examined (0.1 µM) (Figure 6). The induction of ROS was limited by the co-incubation with NAC, a sulfhydryl group containing antioxidant that can act as a ROS scavenger and a precursor of intracellular (reduced) glutathione. Relatively low levels of ROS may not be detrimental to cell health and be functional in physiological processes including cellular signalling and can also be neutralised by the cellular (antioxidant) defence system [46,47]. Nevertheless, a threshold is reached beyond which ROS can bind to and damage cellular components including proteins and lipids, as well as potentially induce genotoxicity via direct or indirect DNA damage [46,47]. Certainly, relatively high levels of ROS are deleterious to the cell and contribute to the induction of apoptosis [47], and apoptosis was confirmed with MQ incubations of 20 µM for 24 h using flow cytometry (Figure 7). This finding matches other studies that have reported that MQ activates caspases to induce cell apoptosis [10,48]. At present, we cannot comment on the type(s) of ROS that are induced in response to MQ, since these cannot be discerned by the DCFDA assay, but this will be of interest to consider in future studies.

The neurotoxicity of MQ and its inadvertent effects on neurophysiology could in part be mediated through influencing neurotransmitter levels and activities. We, therefore, investigated the potential for MQ to act as an inhibitor of acetyl- and butyryl-cholinesterases, the enzymes responsible for the breakdown and cessation of acetylcholine and butyrylcholine signalling at cholinergic synapses. Our novel approach was to undertake in silico molecular modelling to characterize and quantify the binding fit of MQ to active site residues of both cholinesterase proteins (Figure 8, Figure 9 and Figure 10). Interestingly, MQ displayed tighter binding to BuChE than to AChE. This binding of MQ to BuChE (−9.755 kCal/mol) was comparable in affinity to that calculated for the commercial phytochemical drug, galantamine (−10.587 kCal/mol) (results not included), used as a first-line cholinesterase inhibitor treatment for Alzheimer’s disease. It was, therefore, unexpected that from in vitro assays, MQ was a less potent BuChE inhibitor than an AChE inhibitor (Figure 11). 

MQ inhibition of AChE displayed a typical concentration-response curve. Likewise, the level of inhibition of BuChE by MQ also correlated with MQ concentration but was notably lower and only decreased by approximately 10% between 1 and 50 µM, before a more marked inhibition was observed at 100 µM MQ (18%). Other studies have also considered the ability of MQ to inhibit human (recombinant) AChE [16] and reported moderate inhibition (<10% at 10 µM MQ). By contrast, MQ was only a weak inhibitor of AChE of electric eel origin and proposed to be a non-competitive (presumably allosteric) inhibitor [17]. Hence, the more potent inhibition of human AChE by MQ that we report (which is supported by our in silico modelling) could relate to active site binding for the human enzyme rather than that from the electric eel, but we have not undertaken further modelling assessment of the electric eel enzyme. The inhibition of AChE by MQ was detectable at 1 µM (9.8%) and significant at 10 µM (20.5%). Hence, assuming MQ inhibition of AChE in vitro mirrors the concentrations required for cholinesterase inhibition in vivo, then altered and sustained acetylcholine signalling could arise in some patients taking MQ for chemoprophylaxis or anti-malarial treatment, with individual blood levels as high as ≈4500 ng/mL (11.9 µM) [40] and ≈4800 ng/mL (12.7 µM) [41]. 

Our data also showed that MQ was a dual cholinesterase inhibitor able to inhibit AChE and BuChE. However, the concentration-response curves differed with a much slower decline in BuChE activity for the MQ concentrations examined. This reduced inhibition could relate to the strong and stable binding of MQ to BuChE calculated from in silico studies and could, therefore, reflect a low off-rate of MQ from the enzyme, but this will need to be considered with future binding experiments. Preliminary inhibitor studies with MQ suggested that it acted as a non-competitive inhibitor of equine BuChE but in accordance with our in silico data, had a much higher affinity (37-fold lowered Ki) than that for AChE [17]; although, as for AChE measurements, there may be species differences between these results and the human enzyme that we have studied. Agents that act as cholinesterase inhibitors can increase the level and duration of the neurotransmitter ACh in the central and peripheral nervous system and at neuromuscular junctions. Interestingly, rivastigmine, the current Food and Drug Administration (FDA) approved cholinesterase inhibitor that exhibits potent dual acetyl- and butyryl-cholinesterase inhibitor activity [49], has neuropsychiatric side effects that overlap with those for MQ, including anxiety and depression [50], indicative of an influence of cholinergic mechanisms on mood and behaviour.

Commercial mefloquine is administered orally as a racemate (a mixture of the RS and SR isomers (+/− form), with four possible stereoisomers from two chiral centres. The inhibition of AChE and BuChE by MQ has stereospecific preferences [51], and similarly, organophosphorus compounds that bind and inactivate AChE or BuChE through active site binding [30,32] display stereospecific proclivity that influences their inhibitory potential [52]. Hence, the potency of MQ as a cholinesterase inhibitor will likely relate to the enantiomer(s) encountered. Furthermore, the cerebral uptake of MQ and efflux mediated by P-glycoprotein is stereoselective [53]. A preliminary cohort study with healthy male and female volunteers compared the safety and tolerability and pharmacokinetics of the +-MQ enantiomer with the racemate, and this indicated that the +-MQ enantiomer had a more favourable profile for mood and sleep [54]. Hence, MQ could exhibit neurotoxic effects by affecting cholinesterase activity and the associated cholinergic signalling, and these effects may be stereoisomer-specific. MQ also enhances the release of the neurotransmitter GABA through its cholinesterase inhibitor activity [18] and can act as a non-competitive inhibitor of 5-hydroxytryptamine-3 (5-HT3) (serotonin) receptors (IC_50_ of approximately 10 µM) [55], with the potential to influence mood and behaviour. 

## 5. Conclusions

Our data suggest that while chemoprophylactic or therapeutic dosing of MQ is unlikely to trigger neuronal cell loss, it could affect cellular neurophysiology by impacting mitochondrial function and ATP production, induce ROS, and evoke the moderate inhibition of cholinesterase enzymes. Inter-individual variability after dosing results in some patients experiencing relatively high MQ blood concentrations (>10 µM) and there is also the potential for MQ to accumulate within tissues, including the brain. Furthermore, the pharmacokinetic profile of MQ is characterised by a low clearance and a large volume of distribution, and consequently, a long terminal half-life that could promote toxicity, particularly for patients with high dosing regimens and limited metabolism. The consequences of altered cholinesterase activity due to MQ and its impact on other neurotransmitters will need further validation in experimental animals to better understand how it could contribute to neurotoxic sequelae. Lastly, it is noteworthy to consider that if the neurotoxicity of MQ is enantiomer-specific, including the cholinesterase inhibition, then the possibility remains to limit the neurotoxicity and side effects if MQ is produced as a specific unreactive enantiomer if this retained its antimalarial properties. 

## Figures and Tables

**Table 1:** Comparison of MQ toxicity at different concentrations and exposure durations using an MTT assay.

Treatment Concentration (µM)	6 h vs. 24 h	6 h vs. 48 h	24 h vs. 48 h
1	0.0006	<0.0001	NS
10	<0.0001	<0.0001	NS
25	0.0003	<0.0001	<0.0001
50	<0.0001	<0.0001	NS
100	<0.0001	<0.0001	NS

SH-SY5Y cells were treated with MQ, and cell metabolic activity quantified using an MTT assay and results analysed using a two-way ANOVA with Tukey’s multiple comparisons. NS, not significant changes, (*p* > 0.05).

**Table 2:** Comparison of MQ toxicity at different concentrations and exposure durations using an LDH assay.

Treatment Concentration (µM)	6 h vs. 24 h	6 h vs. 48 h	24 h vs. 48 h
0.1	NS	NS	NS
1	NS	NS	0.0234
10	NS	0.0092	0.0045
25	<0.0001	<0.0001	<0.0001
50	NS	<0.0001	<0.0001
100	0.0307	<0.0001	<0.0001

SH-SY5Y cells were treated with MQ, and cell viability quantified using an LDH assay and analysed using a two-way ANOVA with Tukey’s multiple comparisons. NS, not significant changes, (*p* > 0.05).

**Table 3:** Comparison of MQ toxicity at different concentrations and exposure durations using an ATP assay.

Treatment Concentration (µM)	6 h vs. 24 h	6 h vs. 48 h	24 h vs. 48 h
0.1	NS	0.0025	NS
1	0.0398	0.0071	NS
10	NS	NS	NS
15	0.0019	<0.0001	NS
20	NS	NS	NS
25	NS	NS	NS
50	NS	NS	NS
100	NS	NS	NS

SH-SY5Y cells were treated with MQ, and cell viability quantified using an ATP assay and analysed using a two-way ANOVA with Tukey’s multiple comparisons. NS, not significant changes, (*p* > 0.05).

**Table 4:** Comparison of MQ toxicity after 6-, 24-, and 48-h exposures quantified by MTT, LDH and ATP assays.

Duration of Treatment	MTT Assay		LDH Assay		ATP Assay	
	IC_50_	R^2^	IC_50_	R^2^	IC_50_	R^2^
6 h	67.09	0.6291	26.16	0.9079	10.24	0.9224
24 h	23.85	0.9329	16.20	0.8934	4.73	0.8368
48 h	14.23	0.9659	11.68	0.7594	3.19	0.8499

SH-SY5Y cells were treated with MQ, and cell viability quantified using an MTT, LDH, and ATP assay. The concentration of MQ that induced a 50% reduction in cell viability (IC_50_) was estimated using non-linear regression.

**Table 5:** Molecular docking analysis of mefloquine binding to AChE and BuChE enzymes.

Ligand	Docking Score	Binding Affinity (XP-Score)	Glide Energy	Glide-Ligand Efficacy	XP-Hbond	Hydrogen Bonding	Hydrogen Bond Distance (Å)
MQ-4EY5	−8.111	−8.114	−7.018	−0.312	0.000	Tyr-337 O–ligand H	2.1
						Gly-120 O–ligand H	2.4
MQ-6I0C	−9.755	−8.759	−36.623	−0.337	−0.700	Glu-197 O–ligand H	2.2
						His-438 O–ligand H	2.1

**Table 6:** Polar and non-polar residues involved in the interaction of mefloquine within the binding pockets of AChE and BuChE.

	Interaction Type	Residues
MQ-6I0C	Polar	Gly-120, Tyr-337
	Non-polar	Gln-71, Tyr-72, Val-73, Asp-74, Gly-82, Thr-83, Trp-86, Asn-87, Pro-88, Tyr-119, Gly-121, Gly-122, Tyr-124, Ser-125, Gly-126, Ala-127, Leu-130, Tyr-133, Gln-202, Ser-203, Ala-204, Phe-297, Phe-338, Tyr-341, Trp-439, Gly-440, His-447, Tyr-449, Ile-451
MQ-4EY5	Polar	Glu-197, His-438
	Non-polar	Asp-70, Gly-78, Ser-79, Trp-82, Trp-112, Tyr-114, Gly-115, Gly-116, Gly-117, Thr-120, Gly-121, Thr-122, Tyr-128, Ser-198, Ala-199, Pro-285, Leu-286, Ala-328, Phe-329, Tyr-332, Trp-430, Met-434, Met-437, Gly-439, Tyr-440, Ile-442

**Table 7:** Prime MM-GBSA analysis of mefloquine with the enzymes AChE and BuChE.

Ligand	Prime Energy	Ligand Efficiency	Ligand Efficiency Ln	ΔG Bind	ΔG Bind Coulomb	ΔG Bind Solv.GB	ΔG Bind (NS)	ΔG Bind (NS) Coulomb	ΔG Bind (NS) Solv. GB
MQ-6I0C	−22,715.70	−0.337	−2.056	−49.43	−18.42	32.93	−57.93	−18.67	32.99
MQ-4EY5	−22,881.89	−0.312	−1.905	1.37	−48.36	74.21	−25.63	−47.45	74.00

## Data Availability

The data presented in this study are available in this article.
